# Essential Noninvasive Multimodality Neuromonitoring for the Critically Ill Patient

**DOI:** 10.1186/s13054-020-2781-2

**Published:** 2020-03-24

**Authors:** Frank A. Rasulo, Tommaso Togni, Stefano Romagnoli

**Affiliations:** 1 Division of Anesthesiology, Intensive Care & Emergency Medicine, University of Brescia at Spedali Civili Hospital, Brescia, Italy; 2 Department of Health Science, University of Florence, Florence, Italy; 3 Department of Anesthesiology and Intensive Care, Azienda Ospedaliero-Universitaria Careggi, Florence, Italy

## Abstract

This article is one of ten reviews selected from the Annual Update in Intensive Care and Emergency Medicine 2020. Other selected articles can be found online at https://www.biomedcentral.com/collections/annualupdate2020. Further information about the Annual Update in Intensive Care and Emergency Medicine is available from http://www.springer.com/series/8901.

## Introduction

Technology has made huge progress within the field of medicine, where newer and more sophisticated devices have been created to assist clinicians in daily practice. Many of these instruments have become either less invasive or noninvasive. Such is the case for neuromonitoring, where it is now possible to apply multimodality non-invasive monitoring to derive a great deal of information necessary for both therapeutic and prognostic purposes.

Multimodal evaluation becomes paramount when dealing with brain injury, whether it be traumatic or nontraumatic. Such is the case for monitoring of brain stem reflexes, cerebral hemodynamics, and brain function. Brain stem reflexes can be evaluated through a clinical neurological exam. However, this is not always possible, due to drugs or impossibility of evoking the reflexes for inaccessible areas of the scalp or head. Yet, there are reflexes that require a more precise evaluation in order to be useful. Such is the case for the automated pupillary response to light, which can be done with extraordinary accuracy using pupillometry devices. Cerebral hemodynamics, most commonly represented by cerebral blood flow (CBF), intracranial pressure (ICP), and cerebral perfusion pressure (CPP), can now be evaluated with a good level of reliability using brain ultrasound. Cerebral function can be evaluated using electrophysiological monitoring, which has become easily applicable also by neurointensivists, in part due to the development of more user-friendly devices. These three components represent the concept of essential noninvasive multimodality neuromonitoring, which we describe in this chapter.

### Automated Pupillometry

One of the most important parameters to evaluate when performing a clinical neurological examination of the brain stem reflexes is the pupillary light reflex. The pupil constricts when the light signal is carried to the tectal plate in the midbrain, then to the Edinger-Westphal nucleus, and then to the eye where it causes the motor fibers to contract, visualized clinically by pupil constriction. The pupillary light reflex, along with size and size differences between pupils (anisocoria), provides information regarding the functional status of both the optic and the oculomotor nerves.

Until recently, evaluation of the pupils was performed through simple observation of the pupil’s reaction to light evoked by flashlights. Similarly, the pupil’s diameter and anisocoria were assessed by an approximate estimation. However, manual examination of the pupillary light reflex is subject to large inter-examiner discrepancies, as high as 40%, particularly when miosis is present. The discrepancy may be further increased in the presence of other confounding factors such as alcohol, drugs, or hypothermia [1]. Couret et al. observed an error rate of 20% and a 50% failure rate in the detection of anisocoria even for pupils of an intermediate size (2–4 mm) [2]. Larson et al. demonstrated that there was a complete failure in detecting the pupillary light reflex when manual examination was performed when the reflex amplitude was <0.3 mm [3]. The examiner would score the initial diameter of the pupil, followed by light stimulation. Reactivity was described as present or absent, or briskly reactive versus sluggishly reactive.

Recently, automated infrared pupillometry has been introduced into clinical practice, quickly gaining popularity due to its quantitative precision, low cost, noninvasiveness, bedside applicability, and easy-to-use technology, contributing to a modern precision-oriented approach to medicine. With the event of this new technology, it is now possible to add important prognostic and diagnostic information to clinical practice when dealing with the patient with brain injury of various origins.

**Fig. 1:**
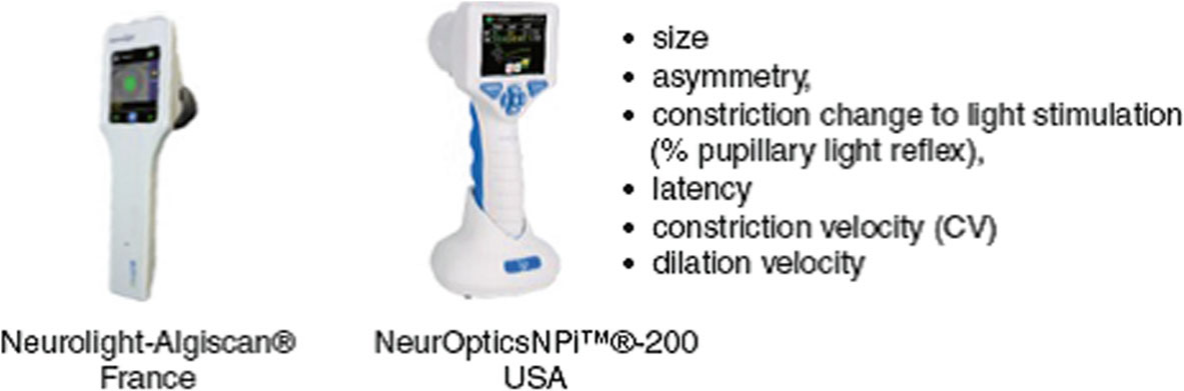
Examples of the automated infrared pupillometry devices available on the market

A few devices are available on the market and are composed of an infrared light-emitting diode, a digital camera that captures the outer border of the iris and senses the reflected infrared light, a data processor, and a screen display showing measured variables in response to the light stimulation, in both a numerical and a graphical format (Fig. 1). The measured variables are size, asymmetry, constriction change to light stimulation, latency, and constriction and dilation velocity. The average reported values are shown in Table 1.

**Table 1:** Parameters provided by manufacturers of two automated infrared pupillometry devices: ^∗^Neuroptics NPi-200 and ^∗∗^NeuroLight-Algiscan

Type of stimulation	Parameters	Normal values
Pupil constriction to light	Diameter (mm)	<0.5 mm
	Asymmetry (mm)	<0.5 mm
	% Pupillary constriction to light (%PLR)	35–40%
	*Latency (s)*	
	Constriction velocity (mm/s)	1.5 mm/s (<1 mm/s: pathological)
	Dilation velocity (mm/s)	2.83
	Neurological pupillary index (NPi)^∗^	≥3
Pupil dilation to pain	Pupillary dilation reflex (%)^∗∗^	33
	Pupillary pain index^∗∗^	Depends on intensity of stimulation [16–19]

*PLR* pupillary light reflex

### Prognosis Following Cardiac Arrest

Clinicians have been checking the pupils of patients with suspected or known brain injury or impaired consciousness for over 100 years. The use of the automated pupillary light reflex has been applied in various forms of brain injury for both prognostic and diagnostic reasons. Its use as a prognostic tool has been mostly studied in the comatose post-cardiac arrest patient. Rossetti et al. showed that bilateral absence of the standard manual pupillary light reflex at day 3 following cardiac arrest was a strong predictor of poor outcome [4]. However, these patients may be under opioid sedation and the pupillary light reflex may be subject to confounding effects, therefore reducing the prognostic accuracy.

Behrends et al. [5] were the first to show that quantitative pupillometry had strong prognostic predictive value during cardiopulmonary resuscitation (CPR) in in-hospital cardiac arrest patients and strong correlations between return to spontaneous circulation and quantitative pupillary were also demonstrated by Yokobori et al. [6]. Pupillometry has been shown to be equally accurate in predicting poor 1-year outcome compared to absent reactivity on the EEG and bilaterally absent N20 waves on SSEPs [5, 6].

One multicenter study recently compared quantitative automated pupillary light reflex and neurological pupillary index (NPi; using the NeurOptics NPi-200, NeurOptics, Laguna Hills, CA) to manual pupillary light reflex in comatose cardiac arrest patients and found that an NPi ≤2, performed between days 1 and 3 following cardiac arrest, was 100% specific for an unfavorable 3-month neurological outcome when compared to manual pupillary light reflex [7].

### Traumatic Brain Injury

Pupillary light reactivity is a well-described prognostic variable in the setting of severe head injury. The literature is full of evidence demonstrating that alterations of the pupillary light reflex, pupil size, and/or anisocoria are correlated with outcome following traumatic brain injury (TBI) [8]. In fact, neurosurgeons triage patients to surgical evacuation of mass lesions or conservative therapy according to the pupillary status [9]. It has also been shown that patients who undergo prompt treatment after a new pupil abnormality, whether it be medical or surgical, have a better outcome [3].

In patients with acute traumatic epidural hematoma and Glasgow Coma Scale (GCS) score <8, anisocoria was present in 67% of patients and reducing the surgery interval to <90 min was associated with a better outcome [10]. TBI patients with a GCS = 3 and fixed, dilated pupils had no chance of survival, whereas patients with a GCS = 3 with pupils that were not fixed or dilated had an excellent survival rate [11]. Intracranial hypertension is associated with decreased NPi, and patients with elevated ICP had an improvement in NPi values after treatment with osmotic therapy. Therefore, pupillometry has the potential as a noninvasive tool to assess the efficacy of osmotic therapy [12].

Stevens et al. performed a prospective observational study on 40 patients with TBI requiring invasive ICP monitoring and showed a weak relationship between ICP events and a preceding NPi event. The strength of this trend appeared to diminish post-decompressive surgery [13]. Jahns et al. assessed 54 patients with severe TBI with abnormal lesions on head computed tomography (CT) imaging who underwent parenchymal ICP monitoring and repeated NPi assessment through four consecutive measurements over intervals of 6 h prior to sustained elevated ICP >20 mmHg and found that episodes of elevated ICP correlated with a concomitant decrease in NPi. Sustained abnormal NPi was in turn associated with a more complicated ICP course and worse outcome [14].

Vassilieva et al. assessed the feasibility of automated pupillometry for the detection of command following in patients with altered consciousness. They enrolled 20 healthy volunteers and 48 patients with a wide range of neurological disorders who were asked to engage in mental arithmetic [15]. Fourteen of 20 (70%) healthy volunteers and 17 of 43 (39.5%) neurological patients fulfilled pre-specified criteria for command following by showing pupillary dilations during 4 or 5 arithmetic tasks.

**Fig. 2:**
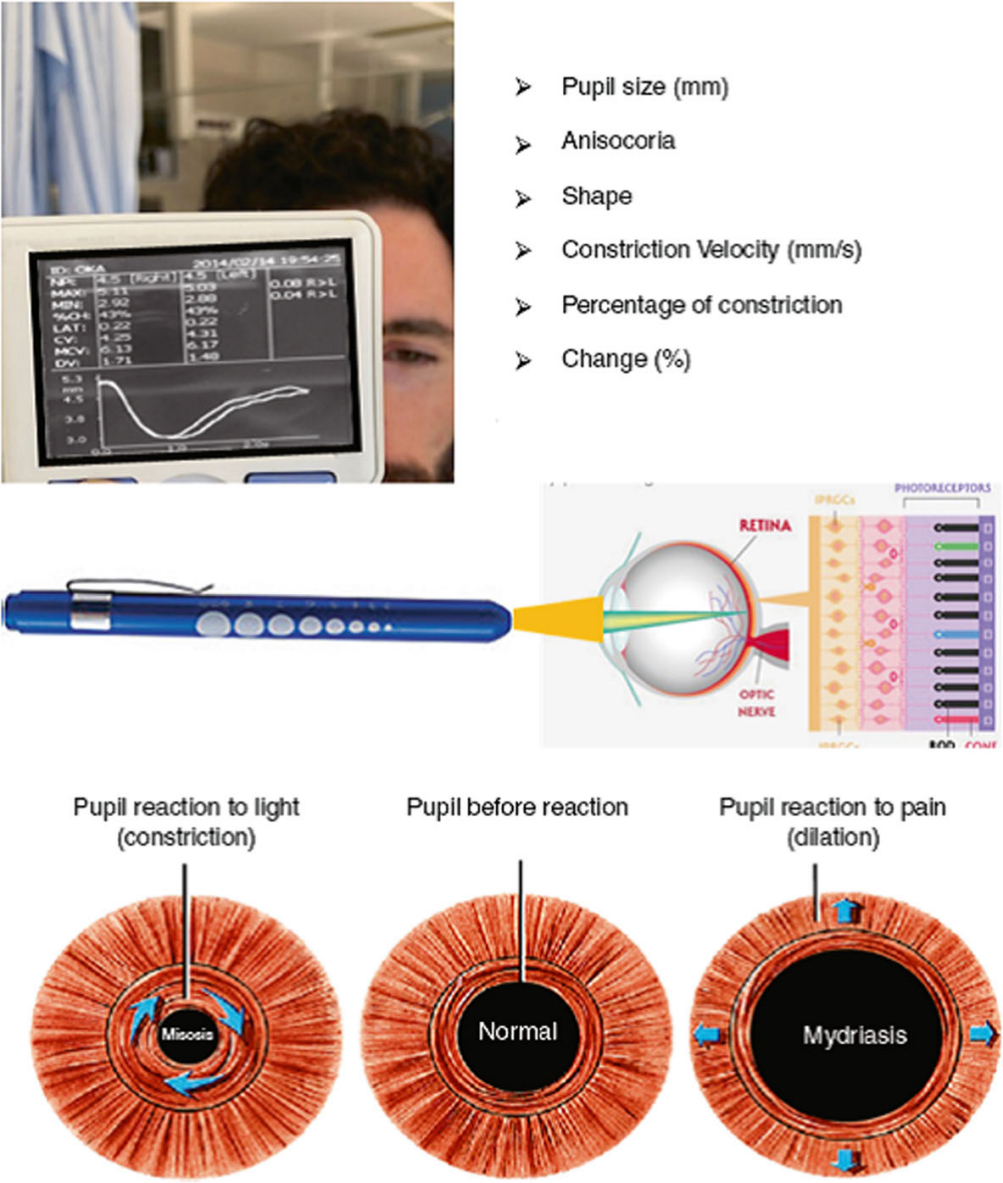
Functions of pupillometry

None of the five sedated and unconscious ICU patients passed this threshold. Therefore, automated infrared pupillometry combined with mental arithmetic appears to be a promising paradigm for the detection of covert consciousness in unresponsive patients with brain injury and may have potential in the future of providing a tool that can reveal covert consciousness in patients in whom standard investigations have failed to detect signs of consciousness (Fig. 2).

### Pain Assessment in Unconscious Patients

Objective nociceptive assessment and optimal pain management have gained increasing attention and adequate nociceptive monitoring remains challenging in noncommunicative, critically ill adults. In the intensive care unit (ICU), routine nociceptive evaluation in mechanically ventilated patients is usually carried out through scales such as the Behavior Pain Scale (BPS). However, this assessment is limited by medication use (e.g., neuromuscular blocking agents) and the inherent subjective character of nociceptive evaluation by third parties.

Since pupillary reflexes are submitted to controlled regulation by the autonomic nervous system, pupillometry allows the assessment of pain in patients subjected to painful stimulation. In fact, pupillary constriction is mediated by the parasympathetic system, whereas dilation is mediated by the noradrenergic sympathetic fibers that are under the influence of stimuli, including stress and pain. A painful stimulus would typically evoke a pupillary dilation reflex. The potential for application of pupillometry for pain evaluation becomes even greater when dealing with the unconscious patient, during general anesthesia for example, where pain assessment scores have no value. Several studies have suggested the use of pupillometry in noncommunicative ICU adults. Paulus et al. demonstrated that pupillary dilation reflex evaluation may predict analgesia requirements during endotracheal aspiration [16]. Moreover, this method may be able to reveal different levels of analgesia and could have discriminatory properties regarding different types of noxious procedures [17]. Recently, scientific interest has been directed toward the use of specific protocols for pupillary dilation reflex assessment because of their low stimulation currents. The pupillary pain index protocol suggested in our approach has been previously investigated in anesthetized adults, revealing a significant correlation between pupillary dilation reflex and opioid administration [18]. Furthermore, Sabourdin et al. demonstrated that pupillary dilation reflex can be used to guide individual intraoperative remifentanil administration and therefore reduce intraoperative opioid consumption and postoperative rescue analgesia requirements [19].

### Brain Ultrasound

Bedside ultrasonography is becoming increasingly widespread in modern medicine, especially in the intensive care setting where this kind of resource is easily accessible and always available to physicians. Brain ultrasonography is a safe, noninvasive way to assess brain anatomy, pathology, and intracranial blood flow. Transcranial Doppler was first introduced in 1982 by Aaslid et al. to record flow velocity in basal cerebral arteries [20]. Advances in technology introduced transcranial color-coded duplex ultrasonography which allows us to assess anatomical features of the brain, rather than just identify brain vessels blindly.

Brain ultrasonography can be applied in different settings, even outside of neurosurgical ICUs: stroke units, enabling physicians to assess the effectiveness of a fibrinolytic therapy, and operating rooms for monitoring CBF during carotid vascular surgery are just some examples of its potential.

Despite being less reliable compared to CT scans and magnetic resonance imaging (MRI), transcranial color-coded duplex ultrasonography is a useful tool to monitor intracranial lesions, such as hematomas, which might cause a midline shift. It might even enable the clinician to assess the ventricles and parenchyma in selected patients with a good acoustic window [21].

### Different Approaches

**Fig. 3:**
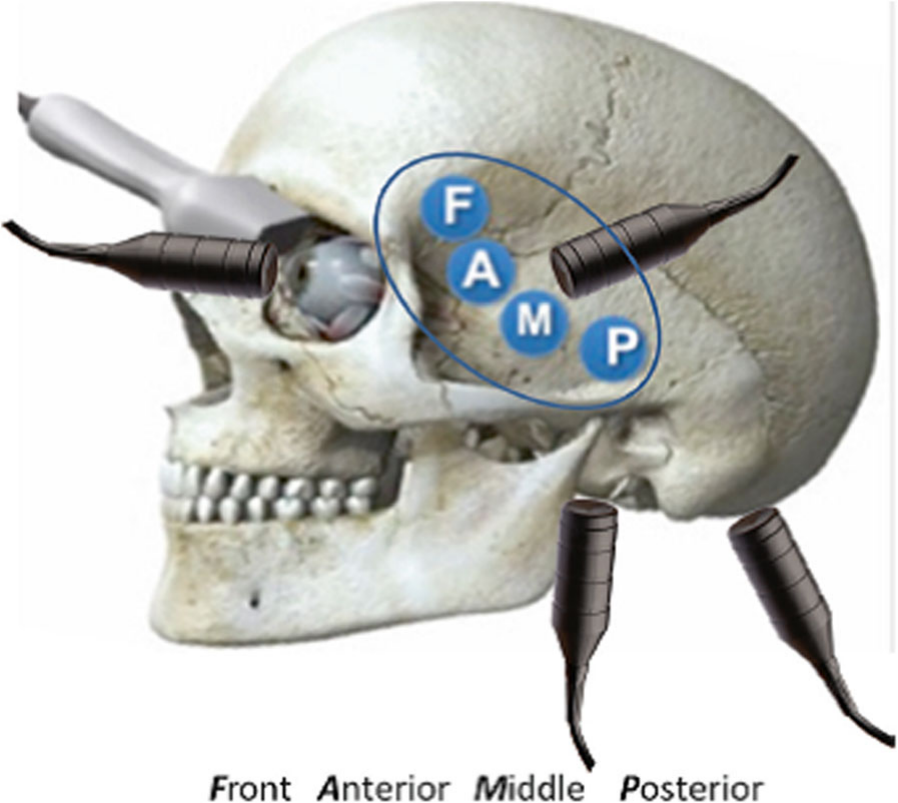
Main approaches to transcranial ultrasonography

**Fig. 4:**
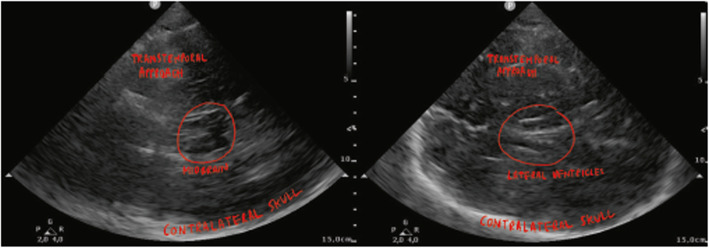
Left panel: Midbrain as the main landmark to explore the circle of Willis; once that is found, the power Doppler can be started to scan for intracranial arteries. Right panel: Lateral ventricles in a severe TBI patient with pronounced midline shift and trans-tentorial herniation

**Fig. 5:**
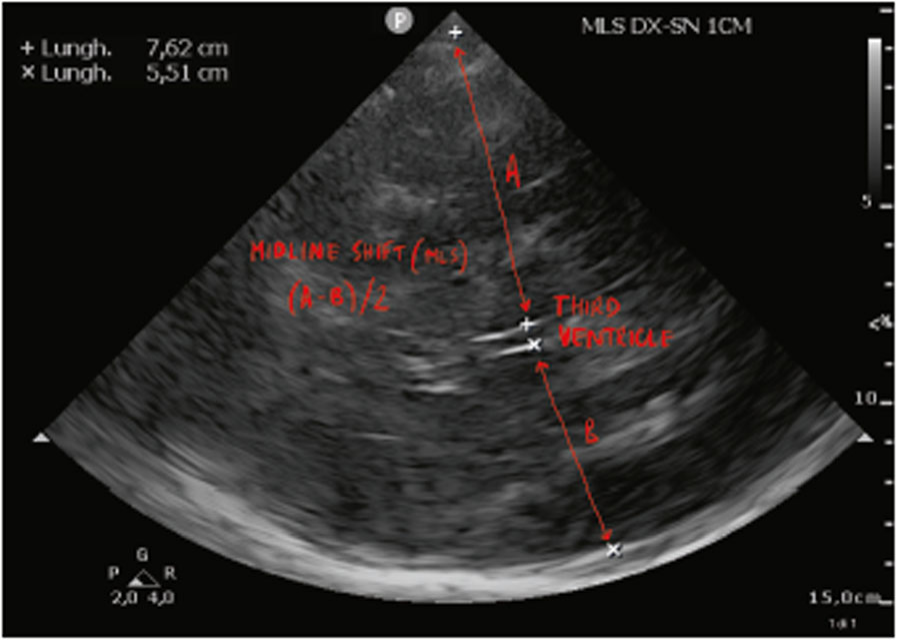
Midline shift in a patient with severe traumatic brain injury. The third ventricle appears as a hypoechoic band between two hyperechoic lines

**Fig. 6:**
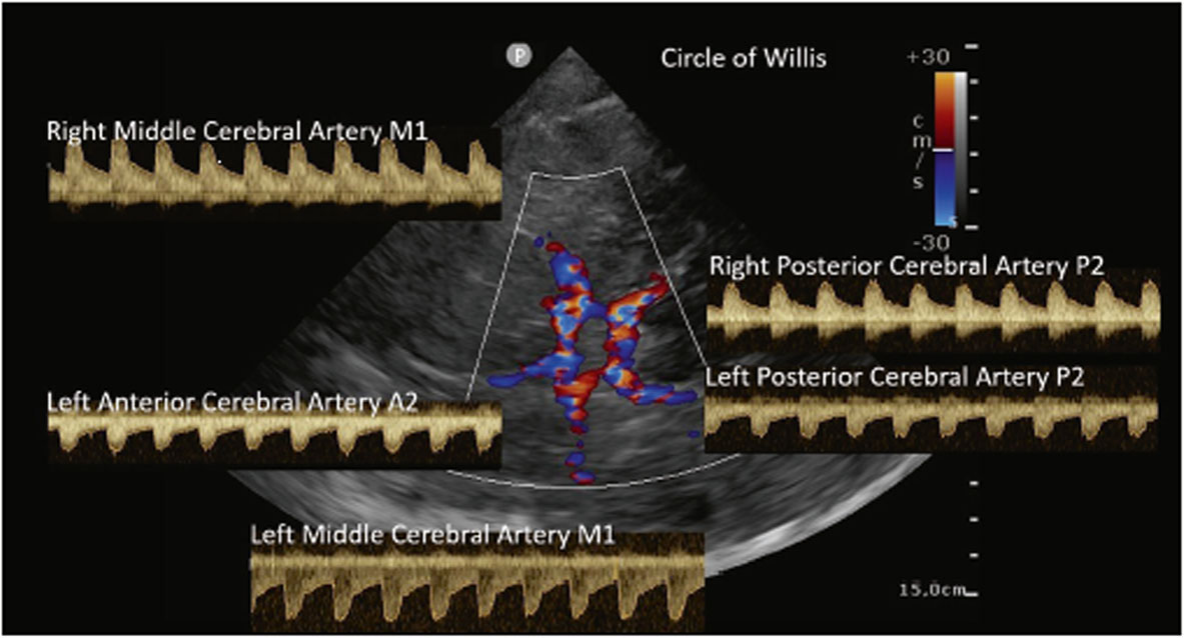
The circle of Willis, as scanned from a transtemporal approach in a patient who underwent a decompressive craniectomy. The different shapes of the arterial flows are shown in the picture

**Fig. 7:**
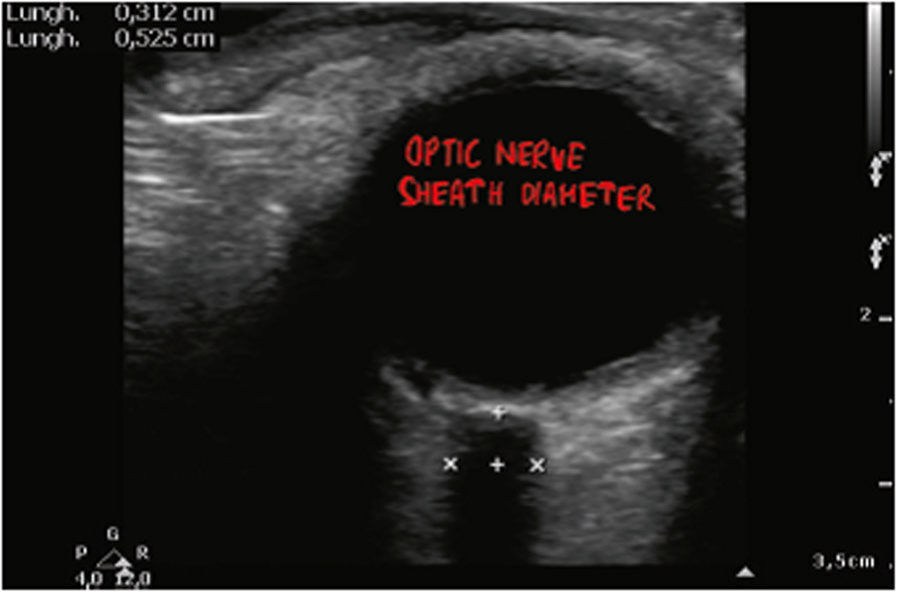
The optic nerve sheath diameter can be measured using a transorbital approach

There are four main acoustic windows accessible for brain ultrasonography, usually performed with a 2–2.5 MHz probe (Fig. 3):

1. Transtemporal approach: between the tragus and the lateral orbit wall, with the probe marker facing toward the eye. The first landmark is the contralateral skull, which is normally around 15 cm deep. The midbrain (Fig. 4 left panel) appears as a hypoechoic shaped heart in the middle of the scan. Once found, the power Doppler can be selected to explore the circle of Willis. This approach is generally used to identify midline shifts (when scanning the third ventricle, which appears as a hypoechoic band between two hyperechoic lines, as shown in Fig. 5) and assess blood flow (Fig. 6).
2. Transorbital approach: Through transorbital ultrasonography it is possible to assess the optic nerve sheath diameter (Fig. 7), as well as blood flow in the ophthalmic artery.

**Fig. 8:**
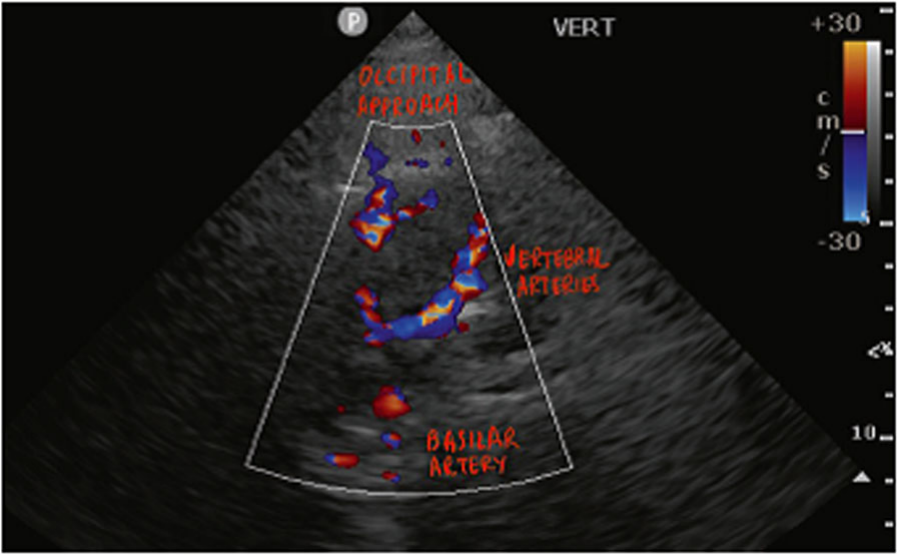
An occipital approach to assess vertebral and basilar blood flow. Landmarks are the hyperechoic clivus and hypoechoic foramen magnum

Occipital approach: The landmark for this approach is 1 cm below the external occipital protuberance, aiming forward and superiorly (toward the eyes), starting with a large scale (11–13 cm); the anatomic landmarks which can be seen with ultrasound are the clivus (hyperechoic structure) and the foramen magnum (hypoechoic). Using the power Doppler function, it is possible to scan for both vertebral arteries ending the basilar artery (Fig. 8).

Submandibular approach: The submandibular window allows assessment of the extracranial and intracranial or extradural segment of the internal carotid artery. The probe should be placed at the angle of the mandible, directed slightly medially and posteriorly. The internal carotid artery can usually be identified at a depth of 40–60 mm.

### The Optic Nerve Sheath Diameter

The optic nerve sheath diameter is a good surrogate measurement for ICP [22]; cutoffs >0.5 cm correlate well with an ICP >20 mmHg. This noninvasive, quick, repeatable way to assess ICP carries a sensitivity of 0.90 and therefore a good level of diagnostic accuracy to quickly detect increased ICP [23]. Using a linear probe placed transversally over the closed eyelid of the patient, the clinician can scan the optic nerve behind the eye, as a hypoechoic structure extending posteriorly from the retina. Measurements of its diameter should be taken 3 mm from the globe perpendicularly (as shown in Fig. 7), using an electronic caliper. The rationale behind this technique is related to the anatomy of the optic nerve, which originates directly from the central nervous system (CNS) and is surrounded by the meningeal sheaths and cerebrospinal fluid (CSF): increases in ICP shift CSF into this space, which increases in diameter. This easy and repeatable technique carries one important pitfall, which is the artifact created by the retinal artery. This vessel runs close to the nerve and might appear as a hypoechoic bump that is particularly difficult to distinguish from the optic nerve. When any suspicion arises, color Doppler mode should be used to evaluate the presence of blood flow.

### Noninvasive ICP Measurement

ICP can be estimated using brain ultrasonography, through a transtemporal approach, assessing blood flow in the middle cerebral artery. The formula used was first introduced by Czosnyka et al. in 1998 [24], originally to estimate CPP noninvasively:

$$ CPP= MAP\times FVd/ FVm+14 $$

where MAP is the mean arterial pressure, FVd the diastolic flow velocity, and FVm the mean flow velocity. However, given that MAP-ICP=CPP, the formula can be written as

$$ ICP= MAP-\left[ MAP\times FVd/ FVm+14\right]. $$

Evidence shows that this method can accurately exclude intracranial hypertension in patients with acute brain injury. The best ICP threshold estimated was 24.8 mmHg, which carried a sensitivity of 100% and a specificity of 91.2% [25]. Another useful tool to consider while estimating ICP using this technique is the pulsatility index. This is calculated as the difference between systolic and diastolic flow velocities, divided by the mean velocity. Many studies have supported the interpretation of the pulsatility index as a tool to reflect distal cerebrovascular resistances, attributing a higher pulsatility index to higher cerebrovascular resistances [26]. However, the pulsatility index is not dependent solely on cerebrovascular resistances, but its value is the result of an interplay between cerebrovascular resistances, CPP, and compliance of the arterial bed. Some authors consider this parameter as less reliable for estimation of ICP [27], and it should therefore be used together with other noninvasive methods for estimation of ICP (transcranial color-coded duplex ultrasonography—optic nerve sheath diameter, as already described).

### Aneurysmal Subarachnoid Hemorrhage and Vasospasm

Vasospasm after aneurysmal subarachnoid hemorrhage (SAH) is the main cause of delayed cerebral ischemia and is associated with severe mortality and morbidity. Guidelines agree on the importance of monitoring blood flow velocities noninvasively [28]. Transcranial Doppler and transcranial color-coded duplex ultrasonography play a pivotal role in the detection of this complication after aneurysmal SAH. Monitoring mean flow velocities is not enough, as an increase in flow velocity does not necessarily imply arterial narrowing. To differentiate this from cerebral hyperemia, Lindegaard et al. [29] introduced a ratio between either the middle cerebral artery or the anterior cerebral artery and the internal carotid artery, using a threshold of 3 as a diagnostic criterion. A Lindegaard ratio of 3 or above was diagnostic for vasospasm, a Lindegaard ratio of less than 3 indicated hyperemia, and a Lindegaard ratio of 6 was highly predictive of severe vasospasm. A blunt increase in flow velocities of 50 cm/s or more within 24 h is also predictive of vasospasm. In 2002, a modified Lindegaard ratio was published for the assessment of basilar vasospasm as a ratio between basilar artery and extracranial vertebral artery, using a cutoff of 2 to differentiate between vasospasm and hyperemia [30].

### Midline Shift

Midline shift can be effectively determined using the transtemporal window, axial plane on ultrasound. The main landmarks are the contralateral skull bone and the mesencephalon, and once those are found and centered in the image, the probe can be tilted cranially 10° until the third ventricle appears in the middle of the scan (diencephalic plane), as two parallel hyperechoic lines in the middle of the field, according to the technique described by Seidel et al. in 1996 [19]. Having identified the third ventricle, the clinician should use an electronic caliper to measure the distance between the ventricle and the inner part of the skull bone, bilaterally. The difference between the two measurements divided by two is the estimation of the midline shift. This relates well with the midline shift measured on CT scan (compared with the Bland-Altman method), regardless of the cause of the shift (spaceoccupying lesion, hematoma) [31] (Fig. 5).

### Cerebral Circulatory Arrest

**Fig. 9:**
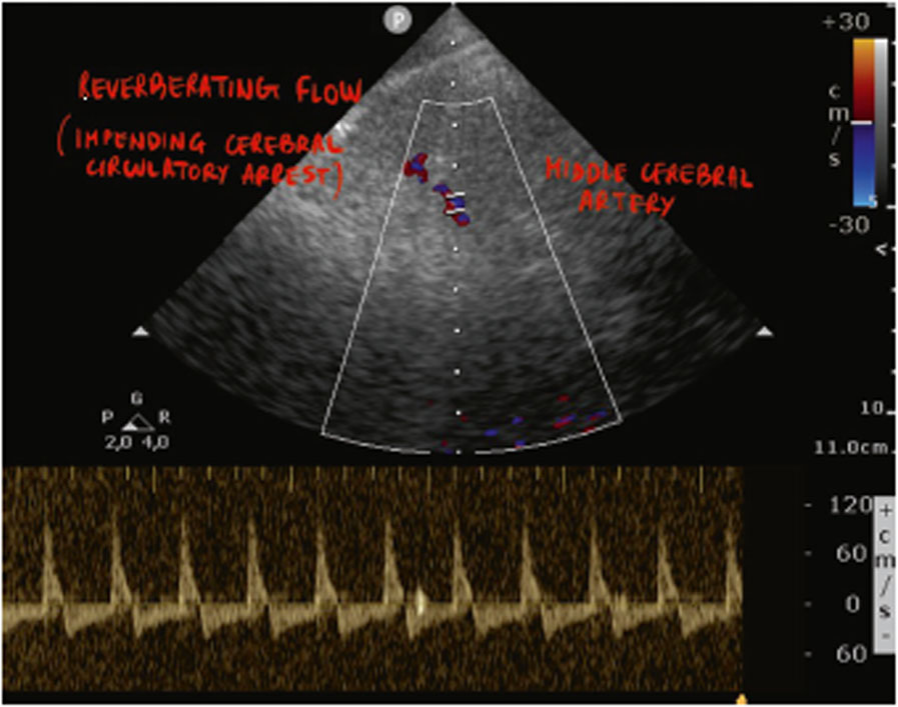
Reverberating flow in a patient with severe brain injury who developed an isoelectric encephalogram trace minutes after this recording and was confirmed brain dead a few hours later

Digital subtraction angiography is considered the gold standard for the confirmation of cerebral circulatory arrest and brain death. However, it requires transport of a hemodynamically unstable patient to the radiology suite to perform an invasive procedure. CBF can be assessed using transcranial Doppler. Increased ICP blunts diastolic flow velocities and, when ICP equals the diastolic arterial blood pressure, flow velocity becomes zero. When ICP increases even further, there is a backflow of blood during the diastolic phase. This phenomenon is called reverberating flow, after diastolic peak blood flows in the opposite direction, and can be assessed with transcranial Doppler. Brunser et al. reported that power mode transcranial Doppler had high sensitivity and specificity for diagnosis of brain death, respectively 100% and 98% (flow velocity was assessed in the middle cerebral artery using a transtemporal approach) [32] (Fig. 9).

### Processed Electroencephalography

Precision medicine represents “a new era of medicine through research, technology, and policies that empower patients, researchers, and providers to work together toward development of individualized care.” With these words, Barack Obama, former president of the United States, launched the Precision Medicine Initiative on January 20, 2015. Funds were dedicated to creating treatments tailored to individual patients’ biologic (genetic and molecular) profiles. Interestingly, current attempts toward standardization of care—protocols, checklists, algorithms, evidence-based medicine, guidelines, consensus papers, and enhanced recovery after surgery programs—challenge precision medicine. While protocols provide guidelines derived from strong evidence that decreases standard variability of care, eventual personalization discovered through clinical algorithms may provide better outcomes [33].

Drug response to sedatives and hypnotics is just one example of interindividual variability related to pharmacogenomics. In this light, identification of the correct dose of sedatives for optimal sedation in the ICU, through proper monitoring and within specific institutional protocols, matches well with the concept of precision and personalized medicine.

**Fig. 10:**
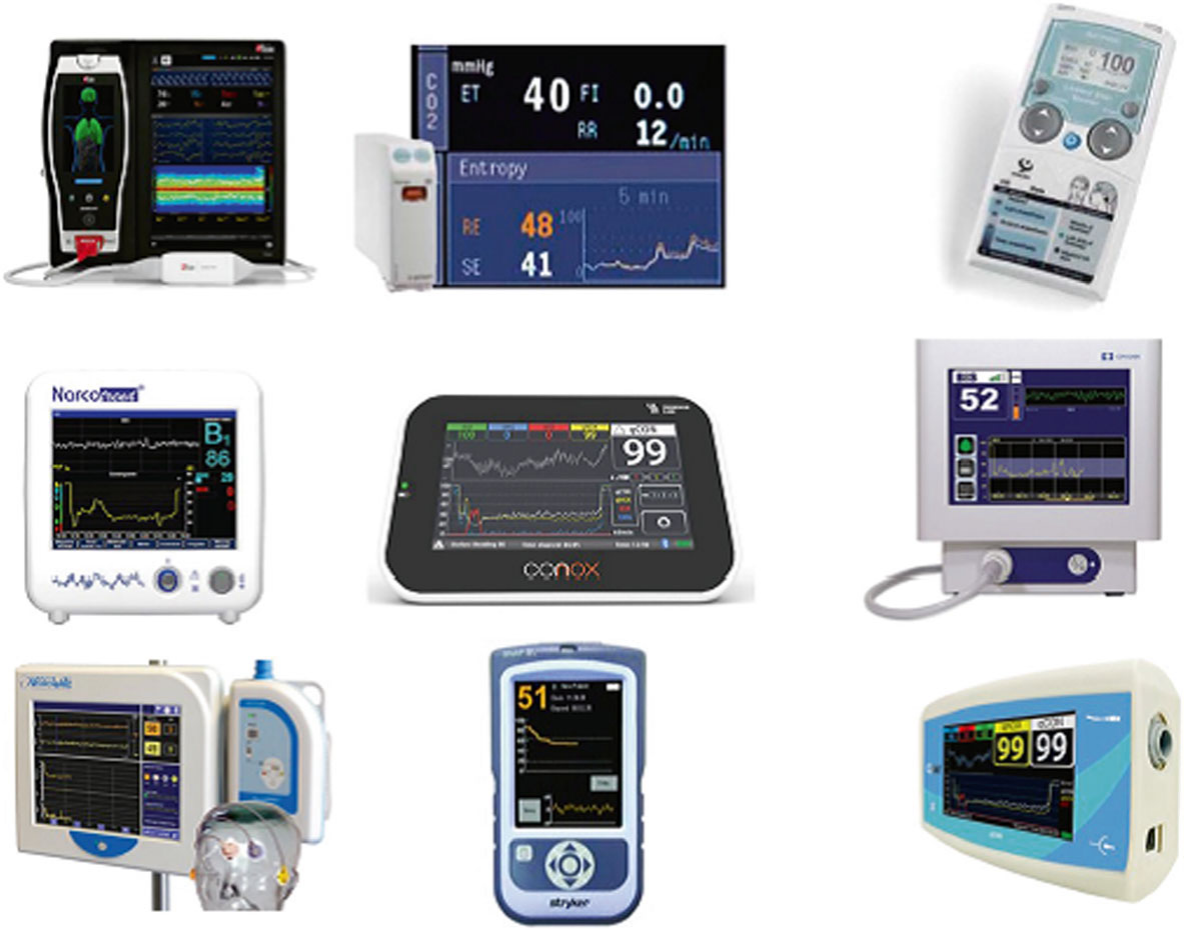
Devices commercially available for processed EEG monitoring

Intensivists continuously monitor their patients’ organs and systems during the ICU stay: of the cardiovascular system using invasive and noninvasive methods; the respiratory system using blood gas and ventilator curve analysis; and renal function using urine output, creatinine, and biomarker levels. The brain is the main target of the sedatives frequently administered to critically ill patients, but no monitor is usually applied to monitor their effect on brain electrical activity, at least outside the neuro-ICU. The main reason for this reality is that electroencephalography (EEG) [34] is a complex investigation system that few intensivists can interpret. Technological evolution has developed a variety of (simplified) EEG-derived indices that can be used to make this information more available. Use of processed EEG indices has been shown to improve intraoperative anesthetic titration during anesthesia but also sedation in the ICU: bispectral index [BIS, Medtronic, Boulder, CO), E-Entropy (GE Healthcare, Helsinki, Finland), Narcotrend (Narcotrend Gruppe, Hannover, Germany), Masimo SEDLine (SEDline, Masimo Corp, Irvine, CA), and NeuroSENSE (NeuroWave Systems, Inc., Cleveland Heights, OH) are a few examples of the tools now available on the market (Fig. 10). There is as yet no evidence for superiority of one device over the others and differences in trace visualization, shape and characteristics of the sensor, institutional habits, and budgets are the main reasons for operator choice [35].

**Fig. 11:**
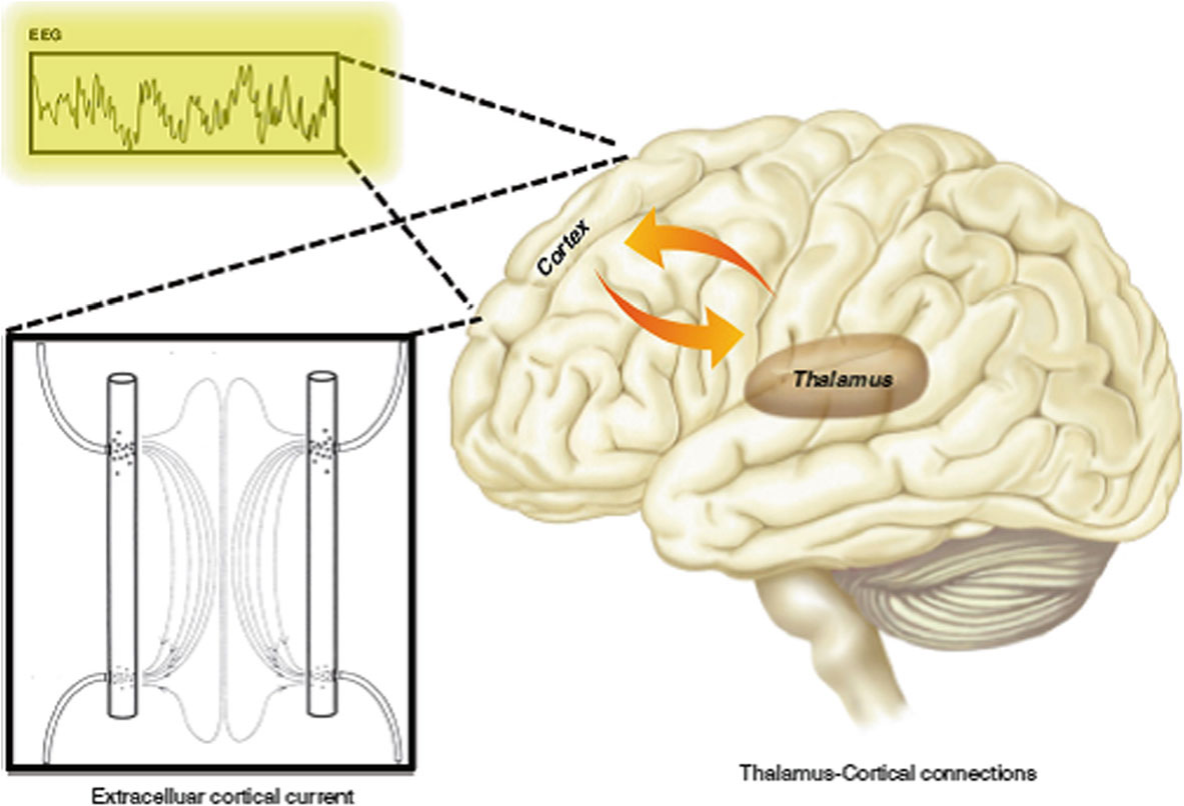
Subcortical-cortical interactions

**Fig. 12:**
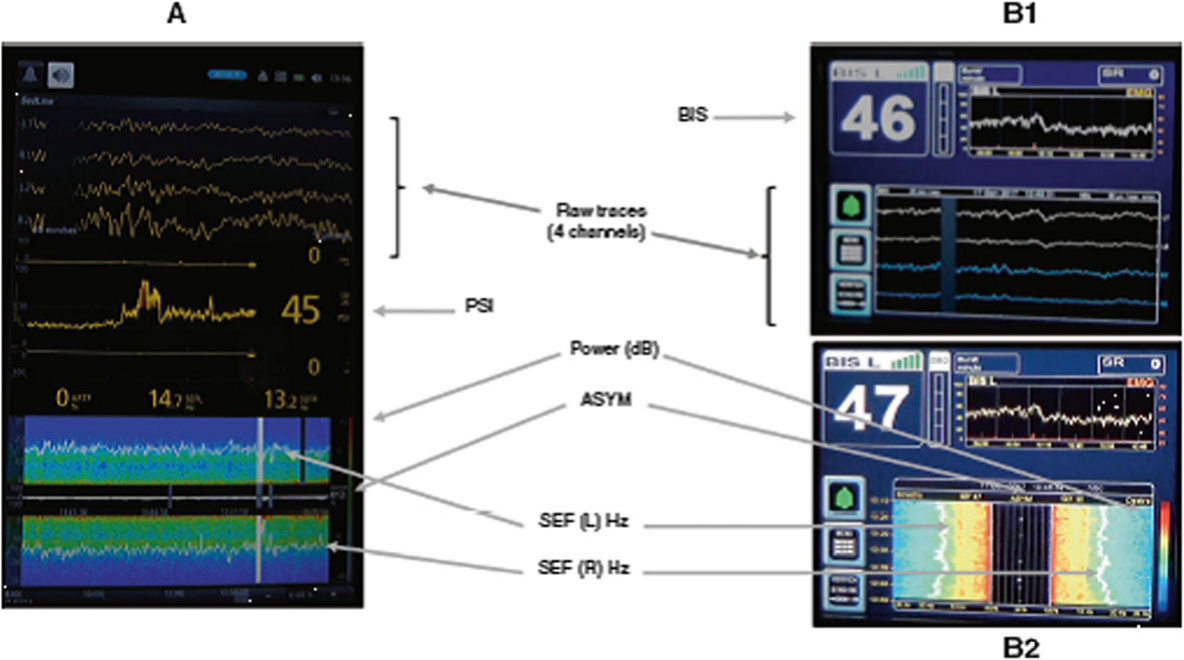
Data delivered by processed EEG devices. A: SEDLine, Masimo Corp, Irvine, CA; B1 and B2: BIS, Medtronic, Boulder, CO. *ASYM* asymmetry, *SEF* spectral edge frequency, *L* left, *R* right

A detailed description of the EEG signal recording and processing is beyond the aim of this chapter, and the reader is referred to dedicated articles [36]. Briefly, subcortical regions (e.g., the thalamus) produce small potentials that cannot be identified from electrodes placed on the scalp because an electric field decreases in strength by the square of the distance from its source (Fig. 11). However, because of the close and continuous interconnection between superficial and deep brain structures, surface EEG reflects the states of both cortical and subcortical areas. Dedicated monitors that automatically elaborate the frontal EEG trace are needed because a full-montage EEG during sedation requires cumbersome equipment and specialized training, not available to all intensivists. Moreover, a frontal processed EEG trace is considered reliable for the purposes of anesthesia/sedation monitoring even if some clinical conditions (see later) eventually require some knowledge of basic EEG principles. Processed EEG monitors deliver three main pieces of information: (1) the raw trace, (2) the numerical index of anesthesia/sedation depth, and (3) the 2D spectrogram (Fig. 12). The reader is referred to dedicated articles for details about the specific parameters [37].

Processed EEG was originally intended for the management of the anesthetic state during surgery to avoid accidental awareness and to titrate sedation in critically ill patients where clinical scales represent the gold standard. The inclusion of processed EEG into many multiparametric ICU monitors reflects the perceived need for ICU caregivers to use a comprehensive approach in the management of sedated patients. Deep sedation is clearly associated with poor short- and long-term outcomes in critically ill patients: prolonged mechanical ventilation and cognitive and psychological complications all increase hospital and ICU length of stay and mortality [38]. Although light sedation, with patients being able to communicate and cooperate at any time, represents a modern target of sedation and a standard of care in ICUs, moderate-to-deep sedation (e.g., a Richmond Agitation–Sedation Scale [RASS] ≤3) may be needed in a non-negligible number of patients, including those with alcohol weaning syndromes complicated by uncontrolled agitation; complex ventilator–patient desynchrony; refractory status epilepticus; intracranial hypertension; patients receiving neuromuscular blocking agents; postsurgical patients requiring hemodynamic, temperature, or bleeding stabilization; post-cardiac arrest therapy (post-resuscitation care); or TBI. In these categories of patients, clinical scales (e.g., RASS, Riker Sedation-Agitation Scale [SAS]), unless they represent standardized assessment of sedation levels, cannot be applied. Moreover, they are commonly evaluated every 4–6 h, and may not detect periods of inadequate sedation occurring between assessments, whereas processed EEG is a continuous method of analysis. In addition, clinical scale assessment is performed by disturbing sedated or sleeping patients (processed EEG does not require modification of the sedation state) and can never identify phases of burst suppression or isoelectric traces (total suppression) [39], which are associated with negative outcomes (e.g., delirium occurrence, prolonged mechanical ventilation, mortality). In this context, in a *post hoc* analysis of a prospective observational study performed in 125 ICU patients under mechanical ventilation, burst suppression occurred in 39% of the cases and was an independent predictor of increased risk of death at 6 months [40]. Processed EEG values can vary greatly in patients sedated in the ICU because, unlike those undergoing painful surgery, patients in the ICU may not experience strong stimulation and therefore require relatively low levels of sedation, appearing calm with BIS values of around 60–80. Clinical procedures, spontaneous patient arousal, physiological sleep cycles, noise, and nursing activities may cause sedation levels to fluctuate. What is important to consider is that muscle activity (mainly) and electric devices (less frequently) may interfere with the ability of the system to process the raw trace, leading to falsely increased sedation indexes [41]. In order to limit this sort of artifact the companies are improving their devices keeping them more “resistant” to EMG interference.

### Recommendations from International Guidelines

In a change from the previous version published in 2013, the recent international guidelines on sedation practice in the ICU [42] (Clinical Practice Guidelines for the Prevention and Management of Pain, Agitation/Sedation, Delirium, Immobility, and Sleep Disruption in Adult Patients in the ICU) report that processed EEG monitoring systems, although best suited for sedative titration during deep sedation or for patients who receive neuromuscular blockade, may also have potential benefits in lighter sedation states and that processed EEG monitoring, compared with the standard clinical scales, may improve sedative titration [43]. Using processed EEG systems as an objective guide for sedative dosing in critically ill patients can decrease the medical complications of oversedation, such as depressed cardiac contractility and hypotension. There are few studies on processed EEG monitoring in the ICU. The first was a prospective trial that randomized patient sedation to be assessed using the Ramsay Scale or BIS monitoring during propofol sedation that was stopped every 2 h [44]. A nurse-guided Ramsey score of 4 was the target in controls, and a BIS value of 70–80 was the target for the study group. A reduction in propofol of 50% was obtained in the BIS group versus controls. The second study [45] was a prospective randomized trial in which patients sedated with morphine and midazolam were randomized to sedation titration based on a BIS >0 versus clinical assessment. No difference was found in the total amount of administered sedative drugs, length of mechanical ventilation, or ICU length of stay. In a recent study on 110 trauma patients, use of BIS resulted in a decrease in sedation and analgesia use, decrease in agitation, less failure to extubate, and fewer tracheostomies, with an approximate 4-day decreased length of stay [46].

Beyond its use for sedative titration purposes, processed EEG may have some additional applications in ICU patients, including identification of subclinical/ unrecognized seizures or seizures occurring when neuromuscular blocking agents are administered. Nevertheless, depending on the frequencies of the ictal waveforms, processed EEG may have variable values that only skilled intensivists are able to read on the raw EEG trace to successfully understand this clinical condition. Processed EEG monitors can also be used to guide therapy aimed at minimizing cerebral metabolism rate to reach predefined levels of burst suppression [47]. A significant proportion of critically ill patients with altered mental status have nonconvulsive subclinical seizures and nonconvulsive status epilepticus [48]. Continuous EEG assessment for nonconvulsive subclinical seizures and nonconvulsive status epilepticus in patients with altered mental status can be indicated in patients with a history of epilepsy, fluctuating level of consciousness, acute brain injury, recent convulsive status epilepticus, stereotyped activity such as paroxysmal movements, nystagmus, twitching, jerking, hippus, and autonomic variability [49]. Nonconvulsive subclinical seizures, seizures with little or no overt clinical manifestations, can be detected with EEG monitoring.

## Conclusion

Noninvasive neuro-multimodality monitoring is now possible. We present an essential bundle of noninvasive neuromonitoring composed of pupillometry, brain ultrasound, and processed EEG. Although some of these noninvasive tools are not yet reliable enough to completely substitute invasive monitoring, they do represent an important adjunct for the clinician in both neuroanesthesia and neurocritical care environments.

We have only described the basic features and the potential that transcranial color-coded duplex Doppler and brain ultrasonography have to offer to the clinician. Bedside ultrasounds are becoming increasingly popular with clinicians because they are quick, reliable, and repeatable. While not yet being a substitute for invasive ICP monitoring, ultrasound can give the clinician useful information when indications for such invasive devices are blurred or contraindicated (liver failure, anticoagulation). Moreover, it has become a mainstay for the early detection of vasospasm in patients with aneurysmal SAH. In the emergency department, expanding focused assessment with sonography in trauma (FAST) assessment to brain ultrasound may enable the physician to become aware of increased ICP even before the patient is transported for a CT scan, and prompt early neuroprotective medical intervention.

EEG is a fundamental tool for monitoring human brain electrical activity during changing states of consciousness like sleep, sedation, or general anesthesia. Processed EEG may contribute to help anesthesiologists and intensivists optimize drug doses in individuals with different pharmacogenomics and clearance of sedatives. Processed EEG devices are not simple plug-and-play units providing a wellinterpretable dimensionless number. They require a global knowledge of technology and of EEG tracings to avoid misinterpretation, especially when muscle activity interferes with the processing algorithm. The use of processed EEG in the ICU could be much more complex than during anesthesia in the operating rooms. Nevertheless, processed EEG monitors offer advantages in the management of patients under moderate and deep sedation and in patients receiving neuromuscular blocking agents to avoid both awareness and burst suppression. Some pathological states, such as seizures or altered EEG states (iatrogenic burst suppression or areflexic coma), may be revealed by processed EEG and trigger a complete EEG examination.

## Data Availability

Not Applicable.
